# The impact of the increase in import verification fees on local production capacity of selected medicines in Uganda

**DOI:** 10.1186/s40545-023-00552-1

**Published:** 2023-03-23

**Authors:** Kalidi Rajab, Solomon Onen, Diana Kesi Nakitto, Allan Serwanga, Joseph Mutasaaga, Leonard Manirakiza, Denis Mwesigwa, David Nahamya, Helen Byomire Ndagije

**Affiliations:** 1 Department of Pharmacy, School of Health Sciences, College of Health Sciences, Makerere University, University Rd., 10218 Kampala, Uganda; 2 National Drug Authority, Lumumba Avenue, 10106 Kampala, Uganda; 3 Uganda National Bureau of Standards, Plot 2-12 By-Pass Link Bweyogerere Industrial and Business Park, P.O Box 6329, Kampala, Uganda

**Keywords:** Local production, Import verification fees, Local industry, Production capacity

## Abstract

**Background:**

The local manufacture of pharmaceuticals is an opportunity to develop a broader manufacturing and knowledge-based economy and reduce over dependence on imports. To promote local production, the Ugandan government introduced Buy Uganda Build Uganda policy geared towards promoting use of locally manufactured goods. It also increased import verification fees in 2017 for 37 selected locally manufactured essential medicines from 2 to 12% to discourage importation of these medicines. This study assessed the impact of the increase in verification fees on local production capacity of the medicines.

**Methods:**

This was a mixed methods study looking at production capacity before and after introduction of the 12% import verification fees. It was conducted among six (6) local pharmaceutical industries in Uganda and seven (7) key informant interviews with experts in the pharmaceutical sector between February and September 2021.

**Results:**

The overall increase in local production capacity of the selected medicines was 8.2% from 2017 to 2020. The most significant increases were in the production of capsules (100.6%, *P* = 0.03) and oral liquids (170.8%, *P* = 0.0001). All the industries registered an increase in number of employees between 2017 and 2020 with an average percentage increase of 42%. There was a 14.7% (95% CI 2.76–17.6%) change in installed capacity of the compression machine (*P* = 0.033) and 27.7% (95% CI 24.6–33.9%) change in installed capacity of the Blow–Fill–Seal (BFS) filling machines (*P* = 0.011). There was also an increase in the number and capacity of installed utilities such as the heating ventilation and air conditioning (968%) and standby generators (131%). Only two (2) industries registered an increase in critical quality control equipment and one had all the critical equipment available by 2020. Most of the key informants reported positive impact of the increment of import verification on local manufacturing capacity.

**Conclusions:**

Local pharmaceutical production capacity increased with the increase in import verification fees with significant increases in production of oral liquids and capsules. Successful implementation of policies supporting local production will promote the development of local pharmaceutical industries. Governments should consider increasing the list of medicines to benefit from the import verification fees increase by adding all essential generic medicines for which there is adequate domestic production capacity and technical skills.

## Background

The access to safe, effective and affordable medicines remains poor in many developing countries [1]. Local pharmaceutical production (LPP) of essential medicines is one of the ways of ensuring the supply of safe, efficacious and affordable medicines. As part of the global development theme to increase access of medicines, over the past decade, LPP has been recognized as an opportunity to increase sustainability and build technical capacity within countries. The intention is to support vulnerable populations especially those in rural areas thus responding to the overarching principle of the 2030 Agenda for sustainable development [2].

LPP is a source of quality assured medicines, contributes to prevention of medicine supplies stock outs, promotes local value addition, reduces medicine costs, generates income by creating jobs, promotes self-reliance and is a step towards sustainability of treatment programs [3]*.* On the contrary, previous reports on the link between LPP and improvement in access to medicines without enabling policy environment remain inconclusive [4]. Nevertheless, the local manufacture of pharmaceuticals in Africa is an opportunity to develop a broader manufacturing and knowledge-based economy [5]. LPP reduces over dependence on imports and international donations and overseas companies who dominate the global market [6].

The capacity for LPP in Uganda and Africa at large still remains low, even though governments have shown political will to support local production because of its benefits. Developing countries, which have three quarters of the world’s population, produce less than 10% of the world's total pharmaceutical output [7]. In Africa, it is estimated that around 79% of all pharmaceuticals are imported [8]. Uganda imports the majority (90%) of the total volume of pharmaceuticals consumed in the country [9, 10]. The Ugandan Pharmaceutical market is mainly dominated by imports from Asia and about 60% of the total volume of pharmaceuticals consumed in the country are distributed by the private sector [11, 12].

To reverse the trend of overreliance on imports and promote local production, countries have either imposed ban on importation or increased import verification fees [13, 14]. Import verification fee is a customs duty-imposed tariff by importing countries on the value of goods brought in from foreign countries. It has been reported that about 60% of all countries levy tariffs on finished pharmaceutical products ranging from 0 to 20% [7]. Further analysis of countries (13%) that levy tariffs between 10 and 20% reveal that they had adequate capacity to locally produce medicines in quantities that satisfy the country’s demands [7].

The Ugandan government introduced Buy Uganda Build Uganda (‘BUBU’) policy geared towards promoting use of locally manufactured goods and use of local skills/personnel [15]. In addition, the government through the Ministry of Health increased import verification fees for 37 selected locally manufactured essential medicines from 2 to 12% effective August 1st 2017 to discourage importation of these medicines and promote local production [16]*.* These selected medicines form part of the essential medicines and health supplies list for Uganda and, fall under either vital (life-saving) medicines, essential or necessary according to the vital, essential, necessary (VEN) classification of medicines [10].

Ever since the introduction of the new policy of 12% increase in import verification fees, there has been paucity of data on the impact of the increment on local production. In this study, we established whether the increase in verification fees for the 37 selected medicines has had an impact on the capacity and volume of local production by the licensed manufacturers. The findings from this study provide evidence on the effectiveness of the policy in promoting local production. This is critical as the country and other developing countries seek to promote local production through increases in import verification fees.

## Methods

### Study aim, design, setting and population

A concurrent mixed methods research design was used to establish the impact of the import verification fees increment on the local production capacity of the affected 37 essential medicines and explore perceptions on the impact of the import verification fees increment on local production of the 37 selected medicines. The list of the 37 selected medicines includes the medicine composition, the dosage forms and strengths, the manufacturer and the dosage form (s) produced by the manufacturers.

The mixed method approach was used to provide a better understanding of the research problem [17]. Quantitative data were collected at the selected local manufacturing facilities using a data extraction checklist, while qualitative data were obtained from key informants using an in-depth interview guide.

By 2019, Uganda had a total of 14 local manufacturers, six (6) of which were manufacturing at least one of the 37 selected essential medicines by the time of the increase of the import verification fees. These local pharmaceutical industries were located in the central region of Uganda in the Districts of Kampala, Wakiso and Mukono.

The study population included key informants from Uganda Pharmaceutical Manufacturers Association (UPMA), Uganda Pharmacy Owners Association (UPOA), Ministry of Health Pharmacy Division, importers of the selected medicines, national central medical stores (National Medical Store and Joint Medical Store) and Ministry of tourism trade and industry. The records of data 2 years before the increase in verification fees (2016/2017) and 2 years after the increase in verification fees (2018/2019) from the local manufacturing facilities were reviewed.

### Selection criteria, sample size determination and sampling procedure

The study included licensed local drug manufacturers in Uganda producing at least one of the 37 selected essential medicines by 2017. No pharmaceutical industry that met the inclusion criteria was excluded. The key informants were purposively selected to participate in the study.

For the quantitative data, the sample size was six (6) pharmaceutical industries. This was a universal sample of all the local pharmaceutical industries producing any of the 37 selected essential medicines.

For the qualitative data, a total of 11 key informants were targeted. However, only seven (7) key informants responded to the interview, and these were key informants from UPMA [3], UPOA [1], importers of the selected medicines [1] and central medical stores [2].

### Data collection methods and tools

Data collection at the local manufacturing facilities included review and extraction of data from factory records using data extraction form. The data collected included human resource capacity, manufacturing capacity, installed equipment capacity, quality control equipment capacity and capacity of installed utilities before and after the introduction of the import verification fees.

An in-depth interview guide was used to collect data from the key informants. Through the interviews, the views and perceptions of the respondents were sought regarding the impact of the increase in verification fees on capacity and volume of local production and any challenges and recommendations regarding the verification fees.

### Data management and quality control

Data were cleaned to ensure that all relevant and correct data were collected. The principal investigator (PI) and study team oversaw accuracy and completeness of all data entered on the checklist before submission for data entry. The data sets entered were cross referenced and errors, and inconsistencies were resolved by checking against the source documents after which one data set was produced. No names were used; identification codes were allocated to each facility checklist. Pre-testing of data collection tools was done in one pharmaceutical industry to ensure that intended responses/meanings for the questions were achieved. The in-depth interviews were audio recorded and transcribed later to ensure that all information given by respondents were not lost. Data collection was done by trained research assistants supervised directly by a member of the lead research team. Computers used for data entry were password protected.

### Data analysis plan

The quantitative data were collected and entered in Microsoft Excel 2017 for cleaning and validation. Analysis was done using Stata version 16. The production capacity was measured by the average quantities produced by local manufacturers before and after the introduction of 12% increase. Paired sample *T* tests was used to test whether there was a significant difference in production capacities before and after 12% increase. Analysis of variance (ANOVA) was used to test the difference in the average quantities produced by local manufacturers.

The qualitative data were transcribed, coded and reported verbatim. Emerging quotes from the interviews were highlighted and marked for referencing.

### Study results

#### Pharmaceutical industry and key informant characteristics

This study involved six (6) pharmaceutical industries and seven (7) key informants. The key informants included members of the UPMA 3 (42.9%), central medical stores 2 (28.6%), UPOA 1 (14.3%) and pharmaceutical importing company 1 (14.3%).

Only one industry manufactures more than 50% (*n* = 31, 83%) of the selected 37 medicines. Majority of the industries manufacture non beta-lactam (*n* = 4, 67%), and oral (*n* = 5, 83%) products (Table 1).

**Table 1:** Characteristics of pharmaceutical industry

Category	Number of medicines/facilities (%)
No of medicines manufactured (*n* = 37)	
Facility 1	4 (10.8)
Facility 2	15 (41)
Facility 3	5 (14)
Facility 4	13 (35)
Facility 5	31 (84)
Facility 6	1 (3)
Scope of products (*n* = 6)	
Both Beta lactam and non beta lactam	2 (33)
Non beta lactam	4 (67)
Scope of certification (*n* = 6)	
Parenteral	2 (33)
Oral	5 (83)
Topical preparations	2 (33)

### Changes in local production capacity before and after the introduction of 12% import verification fees

#### Human resource capacity

All the industries registered an increase in number of employees between 2017 and 2020 with an average percentage increase of 42%. Of the industries, four (4) registered an increase greater than the average and two (2) facilities registered a significant increase (*P* < 0.05) in number of employees (Table 2).

**Table 2:** Number of employees working in the industries

Category	Facilities	2017	2020	Increase in number (%)	*P* value
Number of employees	Facility 1	159	178	19 (12%)	0.34
	Facility 2	249	357	108 (43%)	0.16
	Facility 3	53	85	32 (60%)	0.03
	Facility 4	68	97	29 (43%)	0.16
	Facility 5	310	557	247 (80%)	0.01
	Facility 6	247	273	26 (11%)	0.38
Average no of employees		181	258	77 (42%)	0.18

#### Manufacturing capacity

There was an overall increase of 8.2% in production volume of medicines. Capsules (100.6%, *P* = 0.03) and oral liquids (170.8%, *P* = 0.0001) registered the highest and significant increases in production (Table 3).

**Table 3:** Comparison of production before (2016/2017) and after (2018/2019) the introduction of the 12% import verification fees

Dosage	No. of facilities	Average units (millions) 2016/2017	Average units (millions) 2018/2019	Increase in units (%)	*T*-statistic	*P* value
Tablets	4	6.367	63.300	− 0.366 (− 0.6%)	0.018	0.985
Capsules	2	5.007	10.042	5.035 (100.6%)	2.53	0.030**
Oral liquids	3	2.969	8.040	5.071 (170.8%)	6.87	< 0.0001**
Sterile liquids	2	2.353	3.140	0.787 (33.4%)	1.95	0.057
Sachets	1	15.500	15.972	0.472 (3.0%)	0.034	0.875
Overall average units	6	2.924	31.630	2.392 (8.2%)	0.283	0.778

**Statistically significant at 5% level of significance

**Fig. 1:**
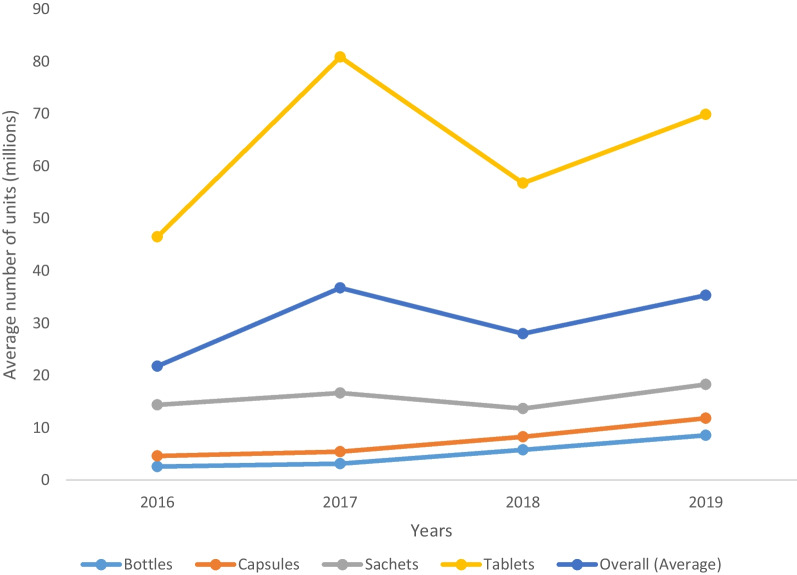
Average units of different dosage forms produced from 2016 to 2019

The production of all dosage forms increased in 2017 and 2019, but there was a drop in production of tablets and sachets in 2018 (Fig. 1).

#### Installed equipment capacity

There were no changes in dry powder and oral liquid/suspension line. Significant changes in installed equipment capacity were observed for compression machine (*P* = 0.033) and Blow–Fill–Seal (BFS) filling machines (*P* = 0.011) (Table 4).

**Table 4:** Installed equipment capacity for granulation/blending line, compression machine, capsulation machine, BFS filling machines and oral rehydration salt (ORS) line

Category	Type of machine	Number of lines	Before June 2017	2020	Change in capacity (CI)	*P* value
Granulation/blending line	RMG	Installed capacity	2400 kg	3470 kg	1.3% [0.85–2.28]	0.167
		Working average capacity	2280 kg	3340 kg		
	FBE/FBD	Installed capacity	2266.7 kg	3058.3 kg	3.1% [0.97–4.87]	0.087
		Working average capacity	2000 kg	2783.3 kg		
	Blender	Installed capacity	1436.7 kg	2766.7 kg	6.0% [0.99–9.86]	0.064
		Working average capacity	1116.7 kg	2280 kg		
Compression machine	Compression machine	Installed capacity	131,875 tabs/h	200,312.5 tabs/h	14.7% [2.76–17.6]	0.033
		Working average capacity	96,750 tabs/h	168,500 tabs/h		
Capsulation machine	Capsulation machines	Installed capacity	30,000 caps/h	49,400 caps/h	6.59% [0.98–8.81]	0.069
		Working average capacity	24,500 caps/h	43,000 caps/h		
BFS filling machines	BFS filling machines	Installed capacity	25,552,824.0	26,970,922		0.011
		Working average capacity	18,966,611.7	25,567,895		
Capacity of oral rehydration salts line	Fitz mill, sifting machine and octagonal blender	Installed capacity	547.6 kg	547.6 kg	27.7% [24.6–33.9]	1
		Working average capacity	547.6 kg	547.6 kg		
	Bossar filling machine	Installed capacity	26,690 sachets for 1 L and 53,170 sachets for ½ L	26,690 sachets for 1 L and 53,170 sachets for ½ L	0.00% [–]	1
		Working average capacity	26,690 sachets for 1 L and 53,170 sachets for ½ L	26,690 sachets for 1 L and 53,170 sachets for ½ L		

#### Inventory of critical quality control equipment

Only two (2) industries registered increase in critical quality control equipment and only one had all the critical equipment available by 2020 (Table 5).

**Table 5:** Availability of critical quality control equipment

Facilities	Proportion of mandatory equipment installed (2017) (%)	Proportion of mandatory equipment installed (2020) (%)	Increase in proportion (%)
Facility 1	76.5	76.5	0.0
Facility 2	81.8	81.8	0.0
Facility 3	87.5	93.8	7.1
Facility 4	94.4	94.4	0.0
Facility 5	95.7	100.0	4.5
Facility 6	85.7	85.7	0.0
Average	86.9	88.7	1.8

#### Capacity of installed utilities

There was a general increase in number (34%) and capacity (493%) of installed utilities. Heating ventilation and air conditioning (HVAC) (968%) and standby generators (131%) registered the highest increases in installed capacity of utilities while decreases were registered for air conditioning (− 37%) and chillers (− 27%) (Table 6).

**Table 6:** Capacity of installed utilities

Utility	Before June 2017		2020		Percentage increase	
	No. of units	Capacity	No. of units	Capacity	No. of units (%)	Capacity (%)
Air conditioning (KW)	NA	1950	NA	1230	NA	− 37
Boilers (Tonnes/h)	7	774.7	7	774.7	0	0
Chillers (KW)	8	5193	8	3785	0	− 27
Compressors (CFM)	7	6462	13	10,667	86	65
Electricity (KVA)	6	8280	7	12,025	17	45
Heating ventilation and air conditioning (CFM)	128	36,837	174	393,587	36	968
Reverse osmosis plant (L/h)	5	10,775	6	13,575	20	26
Standby generators (KVA)	8	3145	12	7265	50	131
Total	169	73,416.7	227	435,643.7	34	493

### Perceptions on the impact of import verification fees increment from 2 to 12% on local production of the 37 selected medicines

Most of the key informants reported positive impact of the increment of import verification from 2 to 12% on local manufacturing capacity. The increment was reported to have caused an increase in employment, expansion especially plants and machinery, production and sales and general increase in consumption of local utilities.

*“Because of this increment, local manufacturers have been able to invest more than before to meet the supply of the 37 products that were made exclusive to them. This has increased the willingness to invest locally. The investment has been mainly in machinery and raw materials KI01”.*

*“The 12% increment in import verification fees increased consumption of locally manufactured products and this has improved the perception of the public to locally manufactured medicines KI02”.*

*“We are now able to get some of these commodities in huge quantities from the local manufacturers save for few other items where we also emphasize BUBU, but some of them cannot produce what is desired for the whole country. By and large, I believe they have the capacity to produce what we need but also for the private sector KI07”.*

Details of the perceptions on impact of the verification fees on local production are summarized in Table 7.

**Table 7:** Perceptions of KIs on impact of 12% increment on local production

Impact on production	Frequency (%), *n* = 7
Improved competitive power of local manufacturers	4 (57.1.7%)
Increased the willingness to invest more locally	3 (42.9%)
It has led to increase in revenue of the local manufacturers	3 (42.9%)
Expanded production capacities of local manufacturers	3 (42.9%)
Encouraged full utilization of resources that were underutilized	2 (28.6%)
Encouraged and attracted investors to invest locally	2 (28.6%)
It has improved on perception of locally produced medicine	1 (14.3%)
There is no expansion at all	1 (14.3%)

### Challenges faced by local manufacturers

A number of challenges were reported by key informants as impeding achievement of the objectives of the increase in import verification fees. Among these were;

1. Export subsidies reduce the market competitiveness of domestic manufacturers. Even with 12% verification fees, some importers were still importing some of the 37 capped products cheaply from countries where export subsidies are provided. Local manufacturers referred to this situation as “dumping,” because the products arrive at a lower price than the locally manufactured products.
2. Non-exclusivity of production of some products, causing investors to lose market concentration. According to the key informants, investors are tempted to go with importation when it appears to be more profitable. As a result, the commitment to using locally manufactured products decreases.
3. The COVID-19 pandemic affected access to raw materials. It was reported that in most cases, raw materials took longer to arrive. This, they claimed, hampered production and resulted in a shortage of some products on the market.
4. Excessive taxes on equipment and spare parts. They stated that, while raw materials are tax-free, they incur high taxation costs of 35–40% for equipment and spare parts.

### Recommendations for improvement from key informants

1. The policy should include all essential drugs locally manufactured rather than limit to only 37.
2. Verification fees should be increased to approximately 22–25% to discourage importation.
3. The local manufacturers should be granted complete exclusivity to manufacture certain products without allowing importation. This can help to mitigate the problem of importer subsidies from the countries of origin. The exclusivity can begin with a few products and then grow over time.
4. Before adding a product to the list of capped products, the capacity of local manufacturers to produce it should be assessed.
5. Government should provide capacity-building grants to small-scale pharmaceutical manufacturers.

## Discussion of results

The findings of this study highlight the changes after the increment in import verification fees on local pharmaceutical production. These provides the necessary evidence on the effectiveness of the policy in promoting local pharmaceutical production. In addition, views from stakeholders provided information not only on the impact of the policy but also broader perspectives on promoting local manufacturing. This evidence is critical as the country seeks to reduce over dependence on imports and international donations and develop a broader manufacturing and knowledge based economy. A study examining tariff rates levied in over 150 countries reported that a number of countries (46%) levy tariffs on finished pharmaceutical products in a range of 0–10% while 13% levy tariffs between 10.1 and 20% to boost local pharmaceutical production [7]. The 12% import verification tax that Uganda levies on the 37 selected medicines falls under the same tariff bracket levied by 13% of the countries who unlike Uganda are in the middle income bracket. Furthermore, by 2012, analysis of countries with tariff rates of 10–20%, revealed that all these countries had the capacity of locally producing medicines in quantities that can satisfy the country’s demand [7].

Overall, local production capacity increased by 8.2% after introduction of the 12% import verification fees. Specifically, there was a general increase in human resource capacity, manufacturing capacity, installed equipment capacity, inventory of critical quality control equipment in some facilities and the capacity of installed utilities. This was corroborated by key informants who reported an increase in employment, expansion especially plants and machinery, production and sales and general increase in consumption of local utilities following the introduction of the verification fees. For the year 2019, according Uganda Bureau of Statistics (UBOS), there was an annual percentage increase of 3.5% in volume of production in the manufacturing sector with chemicals, paint, foams and soap products having the greatest increase of 21.3% [18]. A similar increase of 3% in volume of production was recorded in 2020 and the increase in chemicals, paint, foams and soap products of 14.8% was mainly attributed to increase in production of chemicals and pharmaceuticals (38.3%) [18]. This shows that pharmaceutical production was growing steadily which may be partly due to the policy.

The pharmaceutical industries that manufactures at least five (5) of the products for which import verifications fees were imposed recorded the biggest increases in number of employees. The highest increase of 80% was registered by the facility that manufactures the biggest number of the products. This implies that the verification fees had positive impact on employment, increasing the number of employees. Whereas the study did not establish the technical skills/specialties of the human resource employed during this period, the manufacturers registered an increase in personnel which was one of the reasons the Ugandan government introduced BUBU, to increase local production capacity and use of local skills/personnel [15]. A study on the different strategies to improve production to reach optimum capacity showed that increased capacity was reflected by an increase in the number of employees [17, 19]. Based on the classification of industries by number of employees, four (4) of the manufacturers operate on a large scale (100 or more employees), two (2) operate on a medium scale (31–99 employees) and none was in the small-scale category (6–30) as was the case in 2017. Therefore, none of the manufacturers changed category after introduction of the import verification fees despite increase in number of employees.

The manufacturing capacity increased significantly for capsules (100.6%, *P* = 0.03) and oral liquids (170.8%, *P* = 0.0001), but there was an overall drop in production of tablet dosage forms. These observations were in line with significant increases in installed equipment capacity for compression machines (*P* = 0.033) and BFS-filling machines (*P* = 0.011) that are used while producing these formulations. There was also increase in capacity of capsulation machines though not significant. However, there was no change in capacity of oral liquid/suspension line despite the increased production of oral liquids implying that the industries were previously operating at below capacity. The increase in verification could have created market for oral liquids and the industries maximized the available operation capacity to meet the created demand. Lack of increase in production was attributed by key informants to non-exclusivity of production of the products which tempts investors to go for importation when it seems more profitable. More so, because of COVID-19 pandemic, access to raw materials was limited and the raw materials were taking longer to arrive hindering the production activities and leading to shortage of some products in the market. Other documented challenges hindering local production from literature include; higher costs of production, limited access to affordable business financing, technology, machinery and the associated high skilled expertise from outside Uganda, dependency on importation for active pharmaceutical ingredients (APIs) and almost all excipients and some packaging materials [9]. In addition, high operating cost for manufacturers relying on backup generators and inadequate licensed cold and dry storage facilities to hold APIs prior to final production [9]. The installed capacity of standby generators increased by 131% from 2017 to 2020.

From this current study, local production increased by 8.2% from 2017 to 2020 following the increase in verification fees. Countries such as Ghana and Nigeria imposed a ban on imports of 14 and 18 essentials medicines for which there was adequate domestic production capacity and technical skills to produce, respectively, which boosted their local capacity to meet the country’s demand [13]. In Nigeria, because of the ban, an increase of average annual local production levels in solid dosage forms from about 15–40% was realized. In addition to import bans and tax benefits, local producers have also benefited from other regulatory support and preferential policies from governments as a strategy for promoting local production [14]. Some governments have a local preference policy when procuring medicines, i.e., they will pay more, up to a fixed percentage, for locally produced medicines than for imports. For example in Ethiopia and Tanzania, in awarding tenders, local manufacturers were permitted a preference of up to 25% and 15%, respectively, above an international supplier [20]. In Uganda like Tanzania, the preference margin is 15% [21]. However, without enough capacity to meet aggregate demand, such strategies would prohibitively raise prices of available medicines. In relation to challenges of raw materials, the local industry mostly carries out secondary production and imports most of the raw materials used during manufacture. Interruptions in supply of raw materials will, therefore, affect production of medicines. It would be prudent for governments to promote local production of raw materials in addition to manufacture of finished products to reduce overdependence on importation. Uganda imports close to 90% of its pharmaceuticals including active pharmaceutical ingredients and raw materials which threatens the local pharmaceutical industry and medicine security [9, 10]. Overreliance on imports may lead to a crisis if certain drugs cannot be sourced when required for instance in cases of pandemics-like COVID-19. It is, therefore, important that Uganda embraces the concept of local production for both raw and finished products and government provides necessary support to the local manufacturer.

Installed equipment capacity increased only for compression machines and BFS-filling machines. There was no much increase in critical quality control equipment with only two (2) facilities registering an increase. The critical quality control equipment was determined based on the list of products the facilities manufacture and, therefore, the required quality control equipment to conduct quality control tests. It is important for all the facilities to have the necessary quality control equipment to assure quality of the products that they produce and promote public trust in local products. One of the challenges faced by locally produced products is lack of public trust on the quality of the products. The facility that manufactures most of the products had all the necessary equipment available. It was noted by the key informants that increase in equipment capacity was affected by high taxes levied on equipment and the spare parts of about 35–40%. There was an overall increase (493%) in installed utilities and this could also be partly due to the policy resulting from increased production. Perhaps, if government put into place policies to protect the local manufacturer from high taxation, it would further promote production and increase development of the local pharmaceutical industry.

### Study limitations

It is possible that the COVID-19 pandemic had an impact on the findings, but not significantly, because the pandemic did not affect the majority of the time period 2017, 2018, and 2019. Throughout the COVID-19 pandemic, supply chain operations also continued. We were unable to rule out the possibility that organic growth of local production and other possible cofounders could have had an impact on production capacity. However, this is highly improbable, and our findings are supported by the key informants who attributed the shifts to the increase in import verification fees.

## Conclusions

There was a general increase in human resource capacity, manufacturing capacity, installed compression and BFS filling machines and capacity of installed utilities. Overall, local production increased by 8.2% from 2017 to 2020 with significant increases in production of oral liquids and capsules. There was perceived positive impact of the increment of import verification fees on local pharmaceutical manufacturing capacity.

### Recommendations

1. The 12% verification fees should be maintained and implemented with additional monitoring of production capacity of domestic manufacturers to ensure availability of medicines at all times.
2. The government of Uganda should promote the domestic manufacture of APIs and pharmaceutical excipients in Uganda.
3. The government should impose regulatory policies in terms of standards of skills and production line for local industries intending for their products to benefit from import verification fees in order to achieve availability and cost reduction.
4. The government should evaluate the capacities of local manufacturers to produce a given product before they are added on the list of capped products.
5. Governments should consider increasing the list of medicines to benefit from the import verification fees increase by adding all essential generic medicines for which there is adequate domestic production capacity and technical skills.

### Future studies

1. A study on production volumes to adequately meet the demands of the country and the capacity of the industry to produce the quantities should be conducted.

## Data Availability

The data for the study are available from the corresponding author upon reasonable request.

## Abbreviations

ANOVA: Analysis of variance
API: Active pharmaceutical ingredient
BFS: Blow–Fill–Seal
BUBU: Buy Uganda Build Uganda
CEHURD: Center for Health, Human Rights and Development
COVID: Corona virus disease
HVAC: Ventilation and air conditioning
LPP: Local pharmaceutical production
MOH: Ministry of Health
NDA: National Drug Authority
NMS: National Medical Store
ORS: Oral rehydration salt
PI: Principal investigator
UBOS: Uganda Bureau of Statistics
UPMA: Uganda Pharmaceutical Manufacturers Association
UPOA: Uganda pharmacy owners association
VEN: Vital essential and necessary
